# Trauma-Informed Care in the UK: A Systematic Review and Thematic Synthesis of Qualitative Studies

**DOI:** 10.1192/bjo.2025.10221

**Published:** 2025-06-20

**Authors:** Lotte Scheuner, Isabel Mark

**Affiliations:** St George’s, University of London, London, United Kingdom

## Abstract

**Aims:** Experiences of trauma are highly prevalent within the UK. Within acute psychiatric care, current risk management includes the use of restrictive interventions. Frequent reports of re-traumatisation among service users have sparked debate about its effectiveness and acceptability. Trauma-informed care (TIC) has garnered more attention in recent years as a safer and more acceptable approach, aiming to recognise and respond to trauma in a way which resists re-traumatisation, but there is wide variation as to how this is implemented in the UK at present. The aim of this systematic review is to assess the effectiveness and acceptability of TIC in acute psychiatric care in the UK, and to determine its potential for national implementation.

**Methods:** Five databases (Embase; Global Health; Medline; PsycINFO; Web of Science) were searched for eligible studies between 21/10/24–09/12/24. A total of 2005 studies were found after applying the search terms. Following screening, 12 studies met inclusion criteria; 7 studies from database searching and a further 5 from reference list searching. Qualitative data was analysed and categorised into 7 global themes using thematic synthesis. Quantitative data was summarised in a narrative manner.

**Results:** The following themes were identified: 1) variation in the experiences of staff and service users; 2) barriers to providing psychosocial care; 3) the importance of trauma-informed training; 4) sustainability of TIC; 5) the importance of staff-service user relationships; 6) the importance of a patient-centred approach; and 7) governance and leadership issues. Results showed a decrease in restraint and seclusion incidents post-TIC implementation in acute psychiatric care facilities. Although most service users reported feeling safer and more in control of their treatment, others described feeling forced into reliving their trauma. There was a general consensus that feeling listened to and genuinely cared for by staff helped them to understand their feelings and find ways to address their trauma. Feelings of unpreparedness and unfamiliarity of TIC were common amongst staff. Most agreed that trauma-informed training packages helped them to feel more confident in delivering care.

**Conclusion:** TIC is an invaluable tool for trauma recovery, with existing literature suggesting that it is an acceptable and effective approach to psychiatric care. National implementation of TIC across the UK would likely benefit a large proportion of individuals. However, this study identifies key issues which still need to be considered, including training, sustainability factors, patient involvement, and leadership. Political backing, staff time and resource management would additionally need addressing.

